# Reviewed article: Souza ILB, Brum A, Nardelli A, Baltazar MMM, Matos FGOA, d’Arce LPG. Underreporting of oral cleft cases: a descriptive study, western Paraná, 2015-2019. Epidemiol Serv Saude. 2026:35;e20240716

**DOI:** 10.1590/S2237-96222026v35e20240716.b

**Published:** 2026-03-20

**Authors:** Betine Pinto Moehlecke Iser

**Affiliations:** 1 Universidade do Sul de Santa Catarina, Tubarão, SC, Brazil

## First round comments

Este estudo busca investigar a notificação de casos de fissuras orais tendo como base os dados provenientes

de um centro de referência na região Oeste do Paraná e o SINASC. O estudo é relevante aos serviços do SUS, no entanto carece de informações metodológicas fundamentais para uma melhor avaliação. Desta forma, convidamos os autores a realizar uma ampla revisão no manuscrito, de forma que possa ser reavaliada a sua viabilidade de publicação.

Salienta-se a seguir considerações importantes sobre o manuscrito apresentado:

A comparação de dados de prevalência entre os casos registrados no SINASC e de um centro de referência é de fato relevante, podendo estimar a proporção de subnotificações no sistema, o que de fato parece ter sido objetivo deste estudo, embora não declarado desta forma. Por outro lado, importante avaliar que o centro de referência pode abarcar casos de outras regiões e/ou municípios, por justamente ser um serviço especializado na atenção a estes pacientes. Ou seja, deve-se questionar se: 1) no centro de referência são atendidas apenas pessoas /casos daquela região? 2) qual a população de referência utilizada para o cálculo da prevalência de fissuras no Centro de Atenção e Pesquisa em Anomalias Craniofaciais?

Esses detalhamentos precisam ser incluídos no método do estudo, seção que está muito sucinta, considerando as recomendações do STROBE e check list recomendado pela RESS. Sugere-se descrever, nos métodos, o local do estudo, tipo de atendimento ofertado pelo Centro de referência, de onde são os pacientes, se são atendimentos via SUS... (existe uma informação implícita e sucinta na discussão, página 8, linhas 11-12, sobre ter sido incorporado ao SUS em 2018, ou seja, durante o período analisado, o que também pode ter influenciado os dados?).

Não é explicita, por exemplo, a intenção de estimar a subnotificação do SINASC, embora os resultados e conclusão do estudo salientem isso. Considerando esse propósito, poderiam calcular a sensibilidade / especificidade do sistema, a partir da base do centro de referência.

Também é natural que num centro de referência haja uma maior sensibilidade para detecção de casos, ainda que fissuras orais sejam mais visíveis ao nascimento e, assim, passíveis de identificação e registro, o que deve ser considerado na interpretação dos resultados e discutido pelos autores.

Questões menores:

No título, além de incluir o delineamento do estudo, sugere-se indicar que a análise da subnotificação seja

com base no SINASC.

Resumo: A conclusão do resumo está muito sucinta, ligada a subnotificação do SINASC. No entanto, a forma de avaliação desta subnotificação pode não ter sido a mais adequada, com base nas considerações mencionadas acima. Ainda, como poderia ser resolvida?

Introdução: na página 4/13, linha 34, quando se apresenta o objetivo do estudo, o termo “eficácia” não parece adequado, devendo ser revisto. Os objetivos apresentados devem ser compatíveis aos indicados no resumo.

Métodos: não é indicada a análise da subnotificação, a referência populacional das prevalências, as análises

estatísticas, e os programas para confecção de mapas apresentados (Figura 3).

Resultados: Indica-se nos resultados, página 5, linha 19, um valor de p da comparação de médias de prevalência, o que não foi descrito nos métodos. Seria o teste de Wilcoxon descrito adequado para essa comparação?

Ainda, indica-se no segundo parágrafo dos resultados uma análise de tendência, com base em regressão linear, o que não está indicado nos métodos, e parece não ser o mais adequado considerando apenas 5 pontos no tempo (2015-2019). Sugere-se rever e/ou justificar o seu uso.

Posteriormente, apresenta-se dados descritivos específicos de cidades da região, que podem não ser de interesse do grande público, e sim para um relatório regional e/ou estadual. Sugere-se rever e/ou justificar o seu uso.

Discussão está longa e apresenta trechos confusos, sugere-se rever e buscar objetividade na interpretação dos resultados apresentados. Avaliar se a comparação de dados específicos da regional com dados internacionais é pertinente (por exemplo, página 6/13, linhas 49-55: “A prevalência média anual de fissuras na 10ª Regional de Saúde do Paraná, segundo o SINASC, foi maior que achados posteriores em 2015, 2017, 2018 e 2019 (13)...”

Rever a frase na página 7, linhas 48-51: “Estudos desenvolvidos no estado de Pernambuco reportaram uma taxa de subnotificação de 37,68%, de 67,5% respectivamente, destacando uma maior subnotificação entre as fissuras palatais, atingindo 83,87% (23,24).” A que se refere o respectivamente?

Item Disponibilidade de Dados: No Formulário sobre Conformidade com a Ciência aberta não está preenchido, nem no item específico no texto. Favor rever.

Recommendation: Major revision

## Second round comments

Agradecemos o envio da versão reformulada do manuscrito. Identificamos a necessidade de alguns ajustes ainda, principalmente nos trechos incorporados nesta versão, os quais foram marcados diretamente no texto para facilitar sua identificação. Os principais pontos estão resumidos a seguir:

1. Página 5, linha 95 - Métodos, fonte de dados. No texto indicado, entende-se que foram considerados todos os prontuários do centro de referência. No entanto, os autores descrevem no item anterior que o centro atende pacientes “provenientes principalmente das regiões oeste e sudoeste do Paraná, além de outros estados como Santa Catarina, Mato Grosso do Sul e países fronteiriços, como Paraguai e Venezuela”. Desta forma, sugere-se rever o texto para esclarecer se foram considerados, dentre os atendimentos do centro de referência no período, aqueles cujas mães residem no local de interesse do estudo, ou seja, municípios pertencentes a 10ª regional do Paraná. Colocamos uma sugestão de texto no item, mas fiquem à vontade para escrever da forma que acharem mais adequada para esclarecer esse ponto.

2. Quanto aos métodos estatísticos, inserimos uma complementação na descrição do cálculo da sensibilidade, para deixar mais claro, considerando o público diverso da RESS. Avaliem se estão de acordo.

Na análise de dados, não é citado o uso da regressão linerar para avaliação de tendência (de fato, não parece ser objetivo do estudo), mas posteriormente esta análise é citada nos resultados. Favor rever.

3. Página 5, linha 127: Para ambas as prevalências - com base no SINASC e no centro de referência - o denominador foi o número de nascidos vivos dado pelo SINASC? Importante detalhar.

4. Resultados

4.1 Página 12, figura 2. A figura 2 retrata o número de casos/10 mil nascimentos, ou seja, a prevalência, ou as médias conforme indicado no título? Por favor esclarecer, e rever o título do eixo que indica 100 mil habitantes, diferente do que está indicado nos métodos. Página 6, linha 161: o texto indica “prevalência significativamente maior no Centro de referência quando comparado ao SINASC”, isso no período ou para um ano em específico?

4.2 Na sequência (linhas 164-165), indica-se “Entre os casos do sexo masculino, uma diferença estatística significativa foi observada entre as médias de prevalência”. Esta diferença foi entre o sexo masculino e feminino ou entre SINASC e centro de referência? Favor esclarecer.

4.3 Na figura 2, não aparecem as linhas de tendência geradas, conforme indicado no título. Ainda, questiona-se o uso da regressão linear, não indicada nos métodos, tendo em vista que o objetivo do estudo não foi avaliar tendência (pelo menos não está assim declarado).

4.4 No título das figuras 3 e 4, completar com o local de abrangência do estudo “Centro de referência da 10ª regional...”

4.5 Na descrição da Figura 4, encontra-se que “a maioria das ocorrências de fissuras registradas no SINASC não apresentava CID especificada no sistema, totalizando 14 casos (31,81%)”. Questiona-se: Essa falta de informação ocorreu apenas no SINASC, correto? Não seria de incluir na figura essa categoria ´não informado´?

5. Discussão

5.1 No primeiro parágrafo, indica-se um “Aumento significativo na prevalência das fissuras orais em relação à média paranaense...”. Sugere-se evitar o termo “aumento significativo”, uma vez que não foi realizada uma análise de tendência (não está indicada nos métodos a análise de tendência por regressão linear - acho que de fato não é objetivo do estudo - mas depois esta é citada nos resultados e aqui). Poderia substituir por “observou-se uma maior prevalência...”

5.2 Página 7, linhas 206-2012: rever a escrita do trecho “A prevalência ... mostrou-se superior à de estudos nacionais anteriores, como o estudo de (19) que o analisaram dados brasileiros de fissurados entre 1975 e 1994 observaram-se uma prevalência de 1,9 casos a cada 10 mil nascimento” e completar informações sobre autores ou local do estudo para a citação 19. O mesmo para o estudo 11 indicado na linha 222.

5.3 Linhas 215-217: o trecho ficou confuso “Observa-se que, em comparação com o estudo aqui apresentado, que a região como um todo, a 10ª Regional de Saúde do Paraná apresenta prevalências ainda mais elevadas, tanto nos dados do SINASC quanto do Centro de Referência”. Qual seria o “estudo aqui apresentado”? quando cita “a região como um todo”, refere-se a região Sul ou a 10ª regional de saúde?

5.4 O texto apresentado nas linhas 218-220 (página 7) é similar ao texto das linhas 227-230 na página 8: “uma investigação mais aprofundada para compreender os fatores específicos que contribuem para essas variações regionais nas taxas de fissurados”. Favor rever.

5.5 Sugere-se que o texto indicado na página 8 linhas 265-267 “Os centros de referência especializados no tratamento de fissurados desempenham um papel significativo na coleta de dados epidemiológicos, apresentando uma maior precisão na apuração da totalidade de casos...” seja contextualizado junto ao texto da página 8, linhas 242-246, que indica que “o Centro de Referência não realiza a identificação das fissuras ao nascimento, mas sim recebe casos encaminhados para diagnóstico e tratamento especializado...” e linhas 253-256 “Esses casos não notificados ao nascer não são incluídos posteriormente nos bancos de dados públicos como novos casos de fissurados, mas apenas aparecem nos números relativos, incluídos em atendimentos e cirurgias realizadas no sistema público de saúde”, pois tratam da mesma questão: casos não registrados ao nascimento não aparecem no SINASC, e depois aparecem na assistência, nos centros de referência.

6. Item Disponibilidade de Dados: precisa ser revisto. Em conformidade com a política de ciência aberta, é incorreta a informação “Não se aplica”. Neste item, deve ser informado onde os dados gerados pela pesquisa podem ser acessados pelo leitor.

Informar corretamente a disponibilidade dos dados é requisito de transparência e integridade em pesquisa, previstos nas Política editorial (https://ress.iec.gov.br/p/page/0/peditorial) e instruções aos autores da nossa revista (https://ress.iec.gov.br/p/page/2/instrucoes), e que reproduzimos a seguir:

Política editorial: “Os dados de pesquisa (como bancos de dados, códigos, métodos e outros materiais utilizados ou resultantes da pesquisa) devem, preferencialmente, ser depositados em repositórios de dados confiáveis, como o SciELO Data (lista disponível em https://wp.scielo.org/wp-content/uploads/Lista-de-Repositorios-Recomendados_pt.pdf), com um identificador persistente, como o DOI (Digital Object Identifier).

O acesso a esses dados, por meio de um link para o repositório, deve ser informado na seção “Disponibilidade de dados” e a referência completa deve ser incluída na seção de “Referências”, conforme exemplo abaixo:

Andrade M. Estudo de genes em ratos albinos na América Latina. OSF [dataset], 2018, ASM0000v1. http://dx.doi.org/10.1590/0123-45620187214”

Instruções: “Disponibilidade dos dados do artigo: declaração sobre o acesso aos dados de pesquisa (bancos de dados, códigos, métodos e outros materiais utilizados e resultantes da pesquisa), informar link do repositório e referenciamento, com a devida citação no texto”.

Recommendation: Minor revision

## Third round comments

Para se adequar as normas da RESS e, se aprovado, facilitar a diagramação do seu manuscrito, solicitamos alguns ajustes ainda nesta etapa de análise, conforme segue:

1) Solicitamos que o título da pesquisa seja alterado para indicar primeiro a temática principal do estudo, seguida pelo delineamento, local e período. Uma sugestão seria: “Subnotificação de casos de pacientes com fissuras orais na região Oeste do Paraná: estudo descritivo, 2015 a 2019”.

2) No Formulário sobre Conformidade com a Ciência Aberta, favor adequar o item indicando que “os conteúdos estarão disponíveis no momento da publicação do artigo. Segue títulos e respectivas URLs, números de acesso ou DOIs dos arquivos dos conteúdos subjacentes ao texto do artigo (use uma linha para cada dado): completando com as informações necessárias, além de verificar o link inserido no texto da carta resposta quanto ao item “disponibilidade de dados”, pois não está sendo encontrado: 10.17632/n9pvg96h55.1 e está diferente do apresentado no arquivo pdf, na página 10/15.

3) Resumo: sugere-se incluir na última frase a indicação da edição da ficha “após o nascimento”: “... a qual talvez pudesse ser resolvida, caso houvesse edição dos dados da declaração de nascidos vivos após o nascimento, quando a fissura for detectada”.

4) Página 4, linhas 12-14, na frase “Estudos mostraram que a prevalência de nascidos vivos com fissuras orais no Brasil é de aproximadamente 5,2 a cada 10 mil nascimentos (10,11).” Questiona-se: os dois estudos mostram a mesma prevalência, ainda que de anos diferentes? Considere-se manter apenas o dado mais recente.

5) Um ponto crítico a ser resolvido é a indicação adequada dos aspectos éticos. No quadro de Aspectos éticos, encontra-se a informação de que a aprovação ética deu-se em 29/09/2024, quando nos métodos, página 4, linha 51, indica-se que o estudo fora realizado nos meses de março a julho de 2024, ou seja, previamente a aprovação ética? De acordo com a Resolução 466, item “XI.2 - Cabe ao pesquisador: a) apresentar o protocolo devidamente instruído ao CEP ou à CONEP, aguardando a decisão de aprovação ética, antes de iniciar a pesquisa;”.

A outra questão é que o CAAE e parecer indicados estão citados em outros estudos já publicados, incluindo dois relatos de caso (https://doi.org/10.56238/arev6n2-140; https://bmcmedgenomics.biomedcentral.com/articles/10.1186/s12920-023-01709-2) e, portanto, não relacionado diretamente ao estudo em questão. Solicita-se esclarecimentos, bem como o envio do parecer específico deste estudo.

6) Discussão

6.1 Pagina 7, linhas 32-33: indicar a que se refere a maior prevalência do que a média paranaense, completando a frase “O presente estudo analisou os dados sobre fissuras orais do SINASC, comparando-os com os de um Centro de Referência e observou-se uma maior prevalência das fissuras orais em relação à média Paranaense (na regional de saude analisada?), bem como identificou uma subnotificação das fissuras inseridas no SINASC.”

6.2 Página 7, Linha 39 - ao citar na frase “estudo de (19)”, por favor indicar o nome do autor antes da citação numérica, ou reestruturar a citação. O mesmo vale para a citação das linhas 54-55 “são maiores que a relatada por (11) para o estado do Paraná”.

O texto do segundo parágrafo permanece confuso. Favor ajustar.

Recommendation: Minor revision

